# Parental attachment, attachment to friends, and well-being among Chilean adolescents and emerging adults

**DOI:** 10.1371/journal.pone.0312777

**Published:** 2024-10-30

**Authors:** Omayck Valarezo-Bravo, Mónica Guzmán-González, Anna Włodarczyk, Silvia Ubillos-Landa, Giulia Casu

**Affiliations:** 1 School of Psychology, Universidad Católica del Norte, Antofagasta, Chile; 2 Associate Researcher at Universidad Nacional de Loja, Loja, Ecuador; 3 Faculty of Health Science, University of Burgos, Burgos, Spain; 4 Department of Psychology, University of Bologna, Bologna, Italy; University of Glasgow, UNITED KINGDOM OF GREAT BRITAIN AND NORTHERN IRELAND

## Abstract

Adolescence and emerging adulthood are two life stages marked by notable cognitive and socioemotional changes. During both periods, friendships play an increasingly significant role and can significantly impact individuals positively or negatively. Despite the evolving nature of these relationships, parents remain crucial to the development of both adolescents and emerging adults, playing a key role in their well-being. This study aimed to investigate the mediating role of attachment to friends in the relationship between parental attachment and the well-being of Chilean adolescents and emerging adults. In a cross-sectional correlational study, 363 adolescents (48.2% female) aged 14 to 17 years (M = 15.33, SD = 0.95) and 199 emerging adults (67.8% female) aged 18 to 29 years (M = 22.42, SD = 2.53) completed the Inventory of Parent and Peer Attachment and the Pemberton Happiness Index Scale to assess general, eudaimonic, hedonic, and social well-being. Results of path analyses indicated that, for both adolescents and emerging adults, a more secure attachment to parents was directly associated with higher scores in all well-being dimensions. Additionally, more secure parental attachment was indirectly associated with better eudaimonic and social well-being through a more secure attachment to friends. These findings suggest that enhancing parental attachment could be a valuable focus in clinical interventions aimed at improving overall well-being in adolescents and emerging adults.

## Introduction

Adolescence and emerging adulthood represent critical transitional phases characterized by substantial transformations across multiple spheres of life [1]. Typically spanning ages 14 to 17, adolescence encompasses rapid physical, cognitive, and psychosocial growth, serving as a time of self-exploration and environmental discovery [2, 3]. During this period, adolescents encounter challenges such as physiological changes associated with puberty, the awakening of sexuality, and heightened social and academic demands [4]. Emerging adulthood (EA), which spans ages 18 to 29, is significantly influenced by social and cultural changes, leading to a delay in assuming adult roles. This stage is marked by intense, albeit more focused, identity exploration compared to adolescence [5–8]. EA is also characterized by self-focus, a feeling of being between adolescence and adulthood, instability, and an optimistic view of future possibilities [9]. While these stages involve significant opportunities for growth and maturation, there is evidence indicating an increase in mental health problems and a decline in well-being during both periods [10–17]. Therefore, exploring potential protective factors for well-being is of relevance. One of such factors is the quality of close relationships during adolescence and emerging adulthood.

During these stages, significant changes occur in the relationship with parents [18]; however, the family of origin remains the primary source of support [19, 20]. In fact, the quality of attachment bonds with parents is a well-recognized protective factor for well-being [21–25]. Similarly, as individuals transition from adolescence to EA, peers gain increasing importance, emerging as another crucial protective factor [26–28]. The quality of friendships significantly impacts well-being and psychological functioning [29–32] and plays a fundamental role in the transition to adulthood [27]. Despite evidence documenting the connection between parental attachment, attachment to friends, and well-being, an aspect deserving further analysis is whether attachment to friends can mediate the association between parental attachment and well-being. This hypothesis has been underexplored, with few exceptions [33, 34], and even less so in the Latin American context, where research on well-being from an attachment framework remains limited [16, 35, 36]. This contrasts with the wealth of accumulated evidence in Anglo-Saxon and European countries [19, 31, 37, 38].

Although attachment theory has been established as universal [39], it is also a framework sensitive to culture [40, 41], as specific cultural patterns in terms of family structure and roles may influence the centrality of attachment bonds [42]. Therefore, evaluating whether results obtained in other contexts replicate in a diverse cultural region provides information about their generalizability and helps identify possible particularities. Additionally, analyzing whether attachment to friends is one of the factors accounting for the link between parental attachment and well-being in both adolescence and EA requires further exploration. Although both stages are marked by a series of transformations, the centrality of peers differs in each stage [18, 43, 44]. Examining these questions can generate valuable information to enrich the understanding of well-being and provide practical implications for interventions during two critical life stages.

### Well-being in adolescence and emerging adulthood: The role of parental attachment

Well-being is defined as the state in which a person experiences positive development, satisfaction, and fulfillment in life [45]. Research on well-being traditionally follows two perspectives: hedonic and eudaimonic [46, 47]. Hedonic models, often referred to as subjective well-being, focus on life satisfaction, pleasure, happiness, and the balance of positive and negative feelings [48, 49]. Eudaimonic models, encompassed within psychological well-being, emphasize optimal psychosocial functioning and the development of human potential [50]. Additionally, social well-being, which captures how individuals perceive their roles and functioning within society, adds another dimension to understanding overall well-being [47, 51].

Bringing these perspectives together, Hervás and Vázquez [47] propose a comprehensive conceptualization of well-being that integrates four dimensions: general well-being (overall life satisfaction and vitality), eudaimonic well-being (meaning in life, self-acceptance, personal growth, relationships, perceived control, and autonomy), hedonic well-being (positive and negative affect in daily life), and social well-being (feeling of living in a society that promotes optimal psychological functioning). This multidimensional framework provides a more holistic understanding of well-being, capturing both personal and social aspects of human experience.

Understanding well-being is further enriched by attachment theory, which highlights the role of significant bonds in shaping both individual and interpersonal functioning throughout life [52–54]. Attachment is an innate behavioral system aimed at achieving proximity to a caregiver for security and survival, especially in stressful situations [55]. The interactions with attachment figures foster the development of internal working models (IWMs), which are mental schemas that shape emotions, cognitions, behaviors, and expectations about affective bonds [54, 56]. These are divided into two categories. On one hand, the model of self is characterized by representations of one’s own ability to generate affection, care, and acceptance. On the other hand, the model of others is distinguished by representations of the availability, responsiveness, and reliability of significant attachment figures [57, 58].

Based on this theory, parental attachment is understood as the emotional bond that primary caregivers establish with their children [59]. Bonds characterized by sensitive responsiveness to threat situations, while simultaneously encouraging exploration and autonomy, allow for the development of attachment security, which is characterized by a positive model of self and others [54, 60]. In contrast, attachment experiences characterized by low availability, rejection, or inconsistency in care favor the construction of negative models of self and/or others, resulting in insecure attachment [54]. Thus, attachment to parents during childhood lays the emotional foundation for interpersonal relationships and subsequent personal development [61]. Armsden and Greenberg [59] describe attachment security as comprising the perception of trust (security, respect, and understanding), communication, and low feelings of isolation, anger, and disconnection experienced with the attachment figures.

During adolescence and EA, relationships with parents often undergo significant changes. Adolescents typically desire more personal space and experience decreased emotional intimacy with their parents [62]. As they transition into EA, they gain greater autonomy, often moving out of the family home while continuing to rely on their parents financially [21]. The quality of parent-child relationship often improves during EA compared to adolescence [63]. Despite the evolving nature of these relationships, parents remain crucial to the development of both adolescents and emerging adults, providing essential support and guidance throughout these transitions [64].

The impact of the quality of attachment bonds with parents on well-being is extensively documented [19, 20, 25, 30, 32, 65, 66]. In adolescence, the quality of parental attachment predicts various dimensions of well-being, including academic performance, interpersonal relationships, self-satisfaction, life satisfaction, and positive affectivity [36, 67–69]. Similar findings are observed in emerging adults, where higher levels of attachment security are associated with greater subjective happiness, life satisfaction, and psychological flourishing [21].

Several studies have established that the association between parental attachment and well-being can be understood through a series of mediating variables, such as emotional regulation [70], hope and motivation [71], satisfaction with social support [72, 73], interpersonal communication [74], and self-esteem [36], among others. Additionally, an important factor that might contribute to this association is the individual’s attachment to friends, which plays a significant role in psychosocial adjustment.

### Attachment to friends as a mediator between parental attachment and well-being during adolescence and emerging adulthood

It has been recognized that individuals who have developed secure bonds with their caregivers exhibit similar attachment patterns in romantic relationships and friendships [75]. Considering the increasing importance of peers during adolescence and EA, it is plausible that attachment to friends can serve as a bridge between parental attachment and well-being during these life stages.

From a theoretical point of view, it is expected that the quality of parental bonds shapes the establishment of attachment relationships with friends. When parents have been a source of support and containment, providing a secure base for exploration, it is more likely that positive IWMs of self and others will develop. These models are based on trust in one’s own ability to elicit affection from others, as well as the perceived availability of others [76]. According to attachment theory, IWMs guide the construction of the relational world [76–78]. Therefore, attachment security fosters a sense of agency and competence in managing relationships with others satisfactorily [26]. Similarly, the quality of parental attachment has been linked to the development of various skills, such as academic competence [79], social skills [19], and prosocial behaviors [66]. These skills can contribute to the formation of more secure attachment bonds with friends [19, 66, 79–83] and improve the quality of interactions with peers [84]. The connection between attachment to parents and attachment to friends has been the focus of numerous studies, supporting this association. Attachment security developed from the bond with parents is associated cross-sectionally and longitudinally with attachment security in later stages of life, particularly in relationships with friends [19, 22, 43, 44, 83, 85–88].

In turn, the quality of friendship bonds predicts various well-being indicators, as concluded by a recent meta-analytic review [31]. More specifically, it has been observed that the quality of bonds with friends is associated with well-being indicators such as life satisfaction, loneliness, and happiness in adolescents [89], and with greater psychological well-being and fewer mental health problems in emerging adults [22, 28].

Within the hedonic tradition, two studies have examined the mediating role of attachment to friends between parental attachment and life satisfaction. One study, focused on adolescents, concluded that attachment to friends partially mediated the link between parental attachment and life satisfaction, but this was only evident in females [34]. In another study of emerging adults, only attachment to the mother had an indirect association with life satisfaction through attachment to a romantic partner, while attachment to peers did not emerge as a mediator [33].

### The present study

The current study seeks to evaluate the direct and indirect links between parental attachment and well-being across different domains—including general, eudaimonic, hedonic, and social well-being—in adolescents and emerging adults, by examining their attachment to friends. Our research is guided by the following hypotheses. Based on evidence from several studies linking parental attachment, friendships, and well-being in adolescents and emerging adults [21, 28, 31, 33, 34, 66, 89], we propose that there exists a connection between these factors. Specifically, it is anticipated that stronger attachment bonds with parents and friends will be associated with higher levels of well-being across its various dimensions. Furthermore, given the evidence that parental attachment is linked to attachment to friends [44, 75, 83, 86], and that attachment to friends is connected to overall well-being [28, 33, 34], it is expected that attachment to friends will serve as a mediator in the relationship between parental attachment and well-being.

## Method

### Design and procedure

The present research is designed as a cross-sectional, correlational study. Inclusion criteria were adolescents aged 14 to 17 years old and emerging adults aged 18 to 29 years old. Recruitment was carried out through a combination of social media advertisements and the snowball sampling technique. The data collection took place between September 1, 2022, and December 1, 2023, using an online questionnaire on the Qualtrics platform. Prior to completing the questionnaire, participants were required to review and consent to an informed consent form, which outlined the study objectives and inclusion criteria, emphasized the confidentiality and anonymity of their information, and provided the estimated time needed to complete the questionnaire (30 to 40 minutes). In accordance with ethical guidelines, supplementary informed consent was furnished to the parents of the adolescent participants. The adolescents were provided with the consent forms to take home, and they returned them signed by their parents. The study was approved by the Ethics Committee of the Universidad Católica del Norte, Chile, under N. 031/2022, MAT: Protocol 020/2022.

### Participants

The sample included a total of 562 participants, 363 (64.6%) adolescents (48.2% female) aged 14 to 17 years (M = 15.33, SD = 0.95) and 199 (35.4%) emerging adults (67.8% female) aged 18 to 29 years (M = 22.42, SD = 2.53). The majority of adolescent participants (93.6%, n = 337) were currently enrolled in education, while 6.1% (n = 22) were engaged in both work and studies. The primary activities of the emerging adults included studying (57.8%, n = 115), working and studying (25.6%, n = 51), and solely working (16.6%, n = 33).

### Measures

Perceived attachment to parents and peers was assessed using the 24-item version [90] of the Inventory of Parent and Peer Attachment (IPPA) [59]. This inventory consists of two 12-item scales that assess adolescents’ and adults’ perceptions of the quality of their emotional bonds with their parents and friends, respectively. Each scale addresses three aspects of attachment quality: trust or security (e.g., “When I am upset about something, my parents try to be understanding”, “My friends listen to what I have to say”), communication (e.g., “I tell my parents about my problems and difficulties”, “My friends care about my well-being”), and alienation (e.g., “Talking to my parents about my problems makes me feel embarrassed or stupid”, “I feel lonely or isolated when I am with my friends”). Items were rated on a 4-point scale ranging from 1 (almost never or never) to 4 (always). After reverse keying the alienation items, an overall score can be computed, with higher scores indicating a more secure attachment to parents and friends, respectively. In the present study, internal consistency estimates were adequate for both attachment to parents (*α* = 0.88, *ω* = 0.88) and attachment to friends (*α* = 0.85, *ω* = 0.85).

Well-being was assessed using the Pemberton Happiness Index Scale (PHI) [47], validated in Chile by Martínez-Zelaya et al. [91]. This instrument provides a measure of remembered well-being (retrospective accounts or evaluations of their satisfaction level, happiness, or psychological functioning) as well as a measure of experienced well-being (positive and negative events that occurred the previous day). In this study, only the 11-item remembered well-being scale was used, which addresses general (2 items; “I am very satisfied with my life”, “I have the energy to accomplish my daily tasks”), eudaimonic (6 items; e.g., “I think my life is useful and worthwhile”), hedonic (2 items; “I enjoy a lot of little things every day”, “I have a lot of bad moments in my daily life”), and social well-being (1 item; “I think that I live in a society that lets me fully realize my potential”). Items are rated on an 11-point scale ranging from 0 (totally disagree) to 10 (totally agree). Higher scores in each dimension indicate higher well-being. Reliability estimates in the present study were *α* = 0.86 for general well-being, and *α* = 0.92 for eudaimonic well-being. Due to a null correlation between the two items of hedonic well-being (*r* = -0.04, *p* = 0.38), only one item (i.e., “I enjoy a lot of little things every day”) was used in the present study for hedonic well-being.

### Statistical analysis

Preliminary analyses included Pearson’s correlations to examine the associations between the study variables, and two-way ANOVAs to compare mean scores between groups based on life stage (i.e., adolescence *vs*. EA) and sex. The proposed model including attachment to friends as a partial mediator between attachment to parents and well-being dimensions was tested using path analysis in Mplus 8.4. The maximum likelihood estimation method was employed. Model fit was evaluated using the root mean square error of approximation (RMSEA) and the standardized root mean square residual (SRMR), where values ≤ 0.08 indicate acceptable fit and values ≤ 0.06 indicate close fit, as well as the comparative fit index (CFI), with values ≥ 0.90 indicating acceptable fit and values ≥ 0.95 indicating close fit [92]. A bootstrap procedure with 5,000 samples and bias-corrected 95% confidence intervals (CI) was used to test the indirect effects. The significance of the indirect effects was determined by verifying that the CI did not include zero.

To assess whether the model held consistently across life stages (adolescents and emerging adults), multigroup modeling was used. This involved estimating two models. We first estimated a two-group fully unconstrained model allowing all path coefficients to vary freely across life stage groups. This model served as the baseline for assessing invariance of the relationships between variables. Subsequently, we estimated a fully constrained model, in which all path coefficients were set to be equal across groups. The chi-square difference test between the unconstrained and constrained models was used as a formal test of invariance. Because the fully unconstrained model was a fully saturated model with zero degrees of freedom, the *χ*^*2*^ obtained for the fully constrained model was equivalent to a *χ*^*2*^ difference test comparing the two nested models. A nonsignificant *χ*^*2*^ for the fully constrained model would indicate that constraining the path coefficients to be equal does not significantly worsen model fit, supporting the invariance of path coefficients across life stages. Conversely, a significant *χ*^*2*^ would suggest that adolescents and emerging adults differ in the strength or direction of at least one pathway, requiring follow-up tests on individual constraints to determine which pathways are noninvariant. This approach of comparing fully unconstrained and constrained models is well-established in moderated mediation studies [93, 94], as it effectively minimizes the risk of inflated Type I errors associated with multiple testing [95]. By analyzing the full sample while rigorously testing for differences across groups, this method provides a robust examination of the relationships of interest across life stages and maximizes statistical power.

Statistical significance was set at *p* < 0.05. Interpretation of effect size was based on Pearson’s *r* and standardized path coefficients, with 0.10 considered small, 0.30 medium, and 0.50 large, and Cohen’s *d*, with 0.20 considered small, 0.50 medium, and 0.80 large [96]. The minimal dataset is available as S1 File.

## Results

### Preliminary analyses

Parental attachment correlated positively with attachment to friends, showing a small effect size, and with the well-being dimensions, showing small-to-moderate effect sizes. Attachment to friends had small positive correlations with well-being dimensions, which were largely and positively intercorrelated. The correlations between the study variables are displayed in Table 1.

**Table 1:** Correlations among study variables (*n* = 562).

	1	2	3	4	5
**1. Parental attachment**	-				
**2. Attachment to friends**	0.23***	-			
**3. General well-being**	0.25***	0.11**	-		
**4. Eudaimonic well-being**	0.31***	0.14***	0.84***	-	
**5. Hedonic well-being**	0.25***	0.11*	0.70***	0.76***	
**6. Social well-being**	0.29***	0.14***	0.57***	0.65***	0.55***

* *p* < 0.05.

** *p* < 0.01.

*** *p* ≤ 0.001.

No significant interaction effect was found between life stage and sex in the ANOVAs. Parental attachment scores did not significantly differ based on life stage. Emerging adults were more securely attached to friends than adolescents, with a medium effect size, while no difference was observed between adolescents and emerging adults in well-being. No sex difference was found in attachment to friends, whereas males reported more secure attachment to parents and higher scores in all well-being dimensions compared to females, with small effect sizes. Based on these results, we controlled for sex by including it as a covariate in the subsequent path analyses. Descriptive statistics and ANOVA results are displayed in Table 2.

**Table 2:** Descriptive statistics of study variables and comparisons across life stages and sexes.

	Total (*n* = 562)		Adolescents (*n* = 363)		Emerging adults (*n* = 199)		Main effect of life stage		Males (*n* = 252)		Females (*n* = 310)		Main effect of sex	
	M	SD	M	SD	M	SD	*F*(1,558)	*d*	M	SD	M	SD	*F*(1,558)	*d*
**Parental attachment**	2.71	0.66	2.69	0.65	2.74	0.68	2.02	0.08	2.77	0.65	2.66	0.66	5.61*	0.17
**Attachment to friends**	3.05	0.56	2.96	0.56	3.21	0.53	19.99***	0.46	3.00	0.56	3.09	0.56	1.97	0.16
**General well-being**	5.07	2.94	5.11	3.08	5.00	2.69	0.13	0.04	5.59	2.93	4.65	2.89	13.29***	0.32
**Eudaimonic well-being**	5.29	2.88	5.26	2.94	5.35	2.78	1.00	0.03	5.77	2.82	4.91	2.88	11.68***	0.30
**Hedonic well-being**	5.71	3.36	5.80	3.41	5.54	3.28	0.14	0.08	6.14	3.29	5.36	3.39	5.87*	0.26
**Social well-being**	4.44	3.11	4.43	3.26	4.44	2.83	0.17	0.00	4.89	3.17	4.06	3.02	7.23**	0.27

*M* = mean; *SD* = standard deviation. Score range was 1–4 for parental attachment and attachment to friends, and 0–10 for general, eudaimonic, hedonic, and social well-being.

* *p* < 0.05.

** *p* < 0.01.

*** *p* < 0.001.

### Mediation model

**Fig 1:**
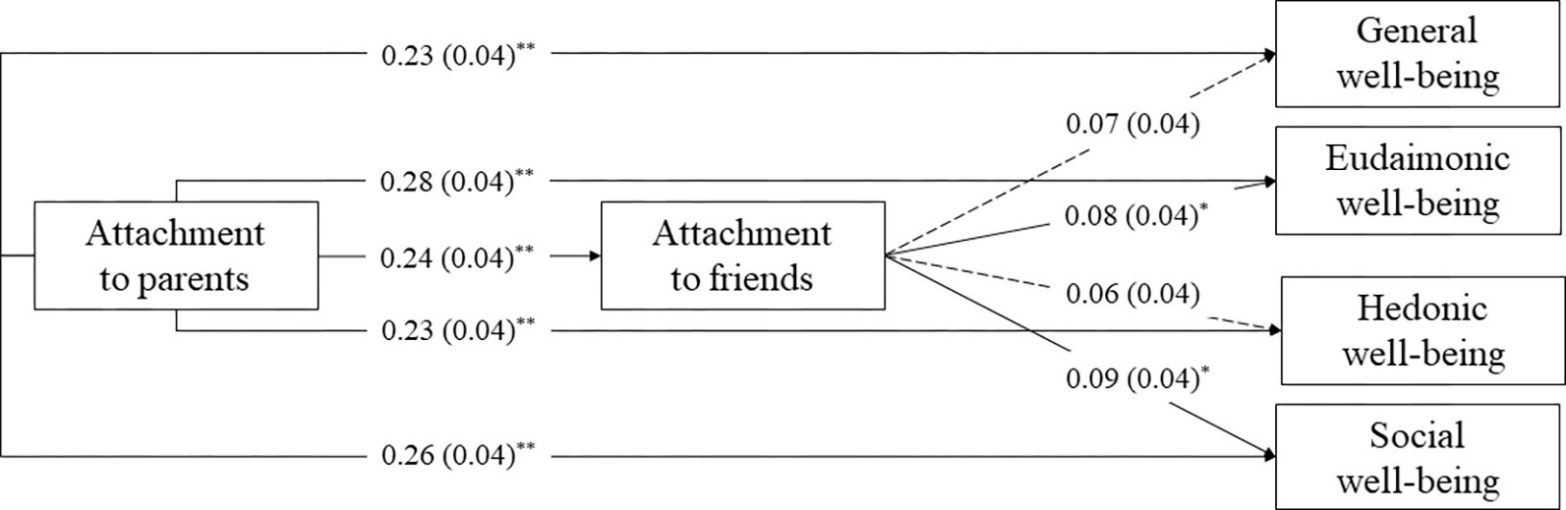
Saturated mediation model in the total sample (n = 562). Standardized estimates (standard errors) are displayed. Correlations between outcome variables and paths from the covariate are omitted for readability. Solid lines represent significant paths; dashed lines represent nonsignificant paths.

In the total sample (*n* = 562), a more secure attachment to parents was significantly, directly associated with slightly higher general (*b* = 0.23, *SE* = 0.04, *z* = 5.18, *p* < 0.001) and hedonic well-being (*b* = 0.23, *SE* = 0.04, *z* = 5.42, *p* < 0.001), as well as moderately higher eudaimonic (*b* = 0.28, *SE* = 0.04, *z* = 6.88, *p* < 0.001) and social well-being (*b* = 0.26, *SE* = 0.04, *z* = 6.33, *p* < 0.001). Parental attachment was significantly, positively associated with attachment to friends, with a small effect size (*b* = 0.24, *SE* = 0.04, *z* = 5.82, *p* < 0.001), and a more secure attachment to friends was significantly associated with slightly higher eudaimonic (*b* = 0.08, *SE* = 0.04, *z* = 2.04, *p* = 0.04) and social well-being (*b* = 0.09, *SE* = 0.04, *z* = 2.05, *p* = 0.04) (Fig 1).

The indirect effects of parental attachment on eudaimonic and social well-being were significant (Table 3). Thus, a more secure attachment to parents was indirectly related to greater eudaimonic and social well-being through a more secure attachment to friends. The model explained 6% of the variance in attachment to friends, and 9%, 12%, 7%, and 10% of the variance in general, eudaimonic, hedonic, and social well-being, respectively.

**Table 3:** Total and indirect effects.

	*b*	*SE*	95% CI
**Parental attachment → General well-being**			
**Total effect**	0.24	0.04	[0.155, 0.323]
**Indirect effect**	0.02	0.01	[0.000, 0.041]
**Parental attachment → Eudaimonic well-being**			
**Total effect**	0.30	0.04	[0.222, 0.379]
**Indirect effect**	0.02	0.01	[0.002, 0.042]
**Parental attachment → Hedonic well-being**			
**Total effect**	0.24	0.04	[0.158, 0.319]
**Indirect effect**	0.01	0.01	[-0.004, 0.037]
**Parental attachment → Social well-being**			
**Total effect**	0.28	0.04	[0.199, 0.355]
**Indirect effect**	0.02	0.01	[0.002, 0.045]

*b* = standardized estimate; *SE* = standard error; CI = confidence interval.

Because the model was a saturated model, including all potential paths among variables and having zero degrees of freedom, no fit statistics could be computed. Following removal of nonsignificant paths to obtain an examination of model fit, the model showed a close fit to the data, *χ^2^*(2) = 3.22, *p* = 0.20, RMSEA = 0.03, SRMR = 0.02, CFI = 0.99.

### Multigroup modelling

Results of multigroup modelling yielded a nonsignificant *χ*^*2*^ for the fully constrained model, *χ*^*2*^(9) = 6.66, *p* = 0.67, indicating that constraining the path coefficients to be equal across life stages did not significantly reduce model fit. This finding supports the invariance of direct associations and pathways from parental attachment to well-being dimensions across adolescents and emerging adults. Since the indirect effects are calculated based on the *a* and *b* paths, and these path coefficients were found to be invariant across groups, it follows that the indirect effects are also consistent between adolescents and emerging adults. Therefore, the consistent relationships observed across groups justify the use of the full sample for mediation analysis, indicating that the indirect effects observed in the combined sample are equally applicable to both adolescents and emerging adults.

## Discussion

The present research aimed to examine whether attachment to friends mediates the relationship between parental attachment and well-being among Chilean adolescents and emerging adults. To achieve this goal, we employed an integrative approach to the well-being construct, considering general, hedonic, eudaimonic, and social well-being. The results obtained partial support the hypotheses of our study.

Regarding the association between parental attachment and well-being dimensions, the findings align with our first hypothesis. As anticipated, a more secure attachment bond with parents was associated with higher general, eudaimonic, hedonic and social well-being in both adolescents and emerging adults. These results are consistent with prior research, supporting the notion that attachment figures provide the foundation for addressing the challenges inherent in these transitional stages, thereby promoting well-being [30, 33, 44, 63, 66, 86, 87]. From an attachment theory perspective, these findings can be explained by several key concepts. Parents offer a secure base that encourage adolescents and emerging adults to confidently explore the world [54, 60]. This secure exploration fosters positive experiences and the development of social and emotional skills, ultimately contributing to enhanced well-being. Additionally, secure parental attachment is linked to better emotional regulation, allowing individuals to manage stress and negative emotions more effectively, which enhances both hedonic and eudaimonic well-being [70]. Importantly, these secure bonds also serve as internal models for future relationships, helping young individuals establish healthy and fulfilling connections with peers and partners, further boosting their social and emotional well-being.

In line with expectations, the association between attachment to friends and well-being was observed in specific dimensions, with greater security in friendships linked to higher eudaimonic and social well-being in both life stages. However, the association with general and hedonic well-being was not significant. These findings suggest that secure attachment to friends is particularly relevant for aspects of well-being related to purpose, personal growth, and social integration. In contrast, more general and immediate aspects of well-being may be shaped by a broader range of factors beyond friendship security, including environmental factors [97] or individual factors such as emotion regulation abilities [70].

Notably, the association between parental attachment and well-being was found to be stronger than that between attachment to friends in both adolescents and emerging adults, which is in line with past studies reporting similar results [34, 98, 99]. This reinforces the idea that while peer relationships gain greater strength and centrality during these stages [26, 100], parental bonds continue to play a crucial role [101]. In fact, several studies consistently support the general claim that parent attachment has a continuous adaptive function throughout childhood and adolescence [26]. Furthermore, longitudinal studies have demonstrated that although individuals develop additional attachment figures during these life stages, their preference for parental attachment remains stable [44, 102].

This dynamic may be particularly pronounced in Latin America, where higher levels of familism are observed compared to other cultural contexts, such as certain regions of Europe (e.g., the Netherlands or Switzerland) [103]. Familism is a cultural value system that prioritizes family unity over individual needs or desires [104]. Within this framework, respect and support are highly valued, and parents are frequently involved in decision-making processes [105, 106]. In times of crisis and distress, the family often acts as a protective factor [107]. This strong cultural emphasis on family may help explain why parental attachment is more strongly associated with well-being than attachment to friends. Despite the growing importance of peer relationships during adolescence and emerging adulthood, the enduring significance of parental bonds in Latin American contexts highlights the crucial protective and supportive role that families continue to play in the well-being of young individuals. Hence, it is possible that while peer relationships gain importance, parental support remains a more reliable predictor of well-being.

In partial support with the study’s central hypothesis, we found evidence of an indirect association between parental attachment and eudaimonic and social well-being through the bond with friends, in both adolescents and emerging adults. Parental attachment bonds characterized by greater trust, better communication, and fewer feelings of alienation were associated with more secure bonds with friends, which, in turn, were associated with higher eudaimonic and social well-being. However, no mediation effect was identified regarding general and hedonic well-being. Thus, while parental and friend attachments are critical for deeper, more enduring aspects of well-being and social competence, immediate and overall well-being might depend on additional variables. Our results are consistent with attachment theory, which posits that the quality of parental bonds shapes attachment relationships with friends [76]. Supportive parents who provide a secure base for exploration help develop positive IWMs of self and others, fostering trust and relationship competence [76]. However, our findings differ from those of Guarnieri et al. [33], where attachment to friends did not serve as a mediator. Among possible explanation for this difference are that Guarnieri’s study distinguished between attachment to mother and father, included romantic attachment as an additional mediator, focused solely on life satisfaction as the outcome, and involved a sample of individuals in a different age range compared to our study.

Understanding the dynamics of attachment to parents and friends offers valuable insights for developing interventions to enhance well-being during adolescence and emerging adulthood, stages marked by increased vulnerability to mental health issues. For instance, developing parenting programs that focus on building secure attachments—through strategies like enhancing trust, improving communication, and reducing feelings of alienation—can effectively promote well-being among adolescents and emerging adults. Similarly, group-based interventions in educational settings should emphasize social skills and peer relationship-building activities to foster supportive friendships, which are crucial for their eudaimonic and social well-being.

Building on this, integrating attachment theory into both educational and clinical settings can further highlight the importance of secure attachment bonds, encouraging practices that not only promote overall well-being but also strengthen resilience and social competence. Specifically, our findings underscore the benefits of fostering secure attachment bonds with parents and friends within clinical practice for the well-being of young individuals. This approach not only aids emotional development but also strengthens resilience and social competence, facilitating more positive adjustment during these critical life stages.

Furthermore, this research provides insights that recognize the distinct nuances observed in studies conducted across different cultural contexts. Future research exploring how cultural factors influence attachment and well-being will deepen our understanding and inform culturally sensitive intervention strategies. Additionally, investigating specific attachment-related interventions and their effectiveness in enhancing different dimensions of well-being should be a priority for future studies.

Building on this, longitudinal studies that track the long-term effects of secure attachment bonds from adolescence through emerging adulthood can identify critical intervention periods. These studies can also examine the relationship between attachment security and specific mental health outcomes, providing valuable guidance for targeted prevention and treatment programs.

However, it is important to acknowledge that our research has limitations that must be considered. Firstly, the cross-sectional nature of the study prevents us from establishing causal relationships. Future studies should utilize longitudinal designs to confirm the temporal relationships between the study variables. Additionally, our study did not differentiate between attachment to mothers and fathers, which is a significant oversight. Attachment to maternal and paternal figures may influence dimensions of well-being differently, as evidenced by research documenting variations in well-being depending on which parental figure is evaluated [28, 33]. We recommend that future research separately assess attachment to mothers and fathers to better understand their distinct impacts on peer relationships and well-being.

Furthermore, the study relied on only one or two items to assess general, hedonic, and social well-being. Future studies should incorporate measures with greater depth and breadth to capture these dimensions more comprehensively. Additionally, the exclusive use of self-report measures introduces the potential for social desirability bias and common method variance. To address this, future research should include multiple data collection methods, such as observational techniques and reports from parents and peers, alongside self-reports. This multi-method approach would help triangulate the data, reduce potential biases, and provide a more thorough understanding of the relationships under study. Finally, given the importance of romantic relationships in emerging adulthood, incorporating attachment to romantic partners as an additional mediator in future models could offer further insights into the dynamics of well-being.

Despite these limitations, the present study contributes to our understanding of well-being in adolescence and EA through the lens of attachment theory. To our knowledge, this is the first study to adopt a more comprehensive approach by incorporating different dimensions of well-being. In summary, our findings indicate that parental attachment has both direct and indirect associations with well-being in both adolescents and emerging adults. These results underscore the crucial role that attachment relationships with parents and friends play in promoting well-being during these life stages.

## Supporting information

S1 File Minimal dataset. (SAV)
